# Estimating body composition using CT scans of cross-bred lambs fed at 2 feeding levels and 2 stages of maturity to inform predictive growth models

**DOI:** 10.1093/jas/skae216

**Published:** 2024-07-30

**Authors:** Thomas P Keogh, Shawn R McGrath, Maxwell B Allworth, Victor H Oddy

**Affiliations:** Fred Morley Centre, School of Agricultural, Environmental and Veterinary Sciences, Charles Sturt University, Wagga Wagga, NSW 2650, Australia; Gulbali Institute, Charles Sturt University, Wagga Wagga, NSW 2650, Australia; CSIRO Agriculture and Food, Black Mountain, ACT 2600, Australia; Fred Morley Centre, School of Agricultural, Environmental and Veterinary Sciences, Charles Sturt University, Wagga Wagga, NSW 2650, Australia; Gulbali Institute, Charles Sturt University, Wagga Wagga, NSW 2650, Australia; Fred Morley Centre, School of Agricultural, Environmental and Veterinary Sciences, Charles Sturt University, Wagga Wagga, NSW 2650, Australia; Gulbali Institute, Charles Sturt University, Wagga Wagga, NSW 2650, Australia; NSW Department of Primary Industries, Livestock Industries Centre, University of New England, Armidale, NSW 2351, Australia

**Keywords:** body composition, growth, modelling, nutrition, ruminant

## Abstract

Livestock producers would benefit from more precise predictions of the growth response from nutrients consumed. Previously published models are often limited by the realities of data collection and are unable to account for alterations to body composition, due in part to the response of visceral organs to an alternate diet. The computerized tomography (**CT**) scanning of lambs enables the analysis of changes in body composition of individual animals over time, potentially supporting better model development and testing. The aim of this experiment was to develop a repeatable method for the analysis of live lamb body composition using CT scans. A secondary aim was to compare the data collected from CT scanning during a feeding trial to 2 predictive lamb growth models. Cross-bred lambs were fed 2 feeding levels at 2 stages of maturity, with CT scans at the beginning and end of each 8-wk feeding period. The CT scan-derived values for body composition taken at the beginning of feeding periods were used as inputs for 2 existing lamb growth models. Predictions of body composition were compared with CT scan-derived values at the end of feeding periods. The CT scan analysis method used a proportion of images from each lamb to reduce manual image editing. The method was developed by comparing the estimated mass and volume of empty body components using all available CT scans to estimated values using a reduced number of scans from 12 lambs. The CT scan-derived lean tissue mass aligned with model predictions at the end of each feeding period, however, CT scan-derived fat mass was greater than predictions by both models especially for the high feeding level at the later stage of maturity. These results highlight that the analysis of body composition using CT scans requires further validation, particularly for the viscera, and that models likely require refinement to better predict the efficiency of energy utilization by different tissues. The use of live animal CT scans can provide more accurate predictions of the growth of saleable products than measuring liveweight alone and will enable ruminant growth models to better adapt to different genetics and changing diets than comparative slaughter. To replicate the current data using comparative slaughter would require 4 times the animals, as individual lambs were CT scanned 4 times in this study, demonstrating the potential value of CT scanning in live animal research.

## Teaser

Tracking changes in individual animal body composition over time increases the availability of data to better inform and develop predictive lamb growth models. The computerized tomography scanning of lambs was used to compare changes in body composition to model predictions during a feeding trial.

## Introduction

The composition of the gain in empty body tissue changes with the animal stage of maturity and a changing feed supply which then alters heat production and the efficiency of energy use for gain (Graham, 1980; Ortigues and Durand, 1995; Sainz et al., 1995; Hegarty et al., 1999). The prediction of body composition by growth models is limited by the requirement to remain as simple as possible with inputs that are easily measured, and the realities of data collection, which typically includes an animal’s liveweight (**LW**) and some estimate of nutrient intake (Oddy et al., 2019). Improvements in the predictions of the effects of genetics, nutrition, and management on body composition, especially of carcass components, and the consequent changes to the energetic efficiency of growth are likely to benefit both producers and processors in the red meat industry (Oddy et al., 1997).

Models of ruminant growth capable of predicting changes in body composition have previously been developed which agree with published data (e.g., CSIRO, 2007; Johnson et al., 2012). However, they do not account for alterations in heat production due to a changing viscera mass (Ortigues and Durand, 1995). Viscera mass is the product of nutritional history and current feed supply and by including information on viscera mass, predictions of weight gain, and body composition are likely to improve (Ferrell, 1988; Burrin et al., 1990).

A ruminant growth model that separates protein mass into 2 separate pools (viscera and muscle) has been proposed and may improve the accuracy of predictions of LW gain and body composition (Oddy et al., 2019, 2024). The muscle pool has a greater mass but lower heat production and the viscera pool has a smaller mass but higher heat production. Fat deposition occurs from the surplus energy available once heat production and protein deposition have been accounted for. Heat production can be predicted by knowing the change in lean body mass and the proportion of that represented by the viscera pool (Oddy et al., 2019, 2024). This approach demonstrates potential for use by producers but is limited by the scarcity of available data on changes in body composition and visceral organ mass with a changing feed supply and animals with different genetics and sexes fed at different stages of maturity.

Data on body composition have traditionally been collected via balance and serial slaughter experiments. These methods are limited by labor requirements constraining the number of animals, and between animal variation because the composition of individuals can only be sampled once (Hegarty et al., 1999). More recently, computerized tomography (**CT**) scanning of live animals has been used to determine carcass composition (Young et al., 1996; Kvane and Vangen, 2006; Macfarlane et al., 2006; Haynes et al., 2010) and estimate rumen volume (Haynes et al., 2012; Bain et al., 2014; Goopy et al., 2014; De Barbieri et al., 2015; Bond et al., 2017).

It is hypothesized that multiple CT scans of the same individual can be used to inform predictive growth models. The first aim of the current research was to analyze CT scans of lambs and develop a repeatable method to separately analyze changes to both carcass and viscera composition. A secondary aim was to compare the predictions of published growth models, referred to as the CSIRO and Oddy models (CSIRO, 2007; Oddy et al., 2024), with body compositional data derived from live animal CT scans during a feeding trial. The experimental data used originated from the experiment reported by Keogh et al., (2023).

## Materials and Methods

### Experimental animals and design

The use and care of animals were approved by the Charles Sturt University (**CSU**) Animal Care and Ethics Committee (protocol number A20203).

A lamb feeding experiment was conducted from October 2020 to May 2021 at the CSU Lamb Feeding Facility, Wagga Wagga (30°03ʹ30.3ʺS, 147°20ʹ38.5ʺE; 219 m altitude). Mixed sex (female and castrated male) cross-bred lambs (progeny of Merino × Border Leicester ewes joined to White Suffolk and Poll Dorset rams, *n* = 108) were sourced from a commercial farm and fed in 36 pens, each pen containing 3 lambs (Keogh et al., 2023). Half of the pens (*n* = 18) were allocated to each treatment and fed either a low (L) or high (H) feeding level of a pelleted diet; approximately 2.5% and 3.5% of LW in dry matter (**DM**), respectively. The pelleted diet comprised predominantly barley grain (47%), lucerne hay (20%), and cereal hay (20%) and contained 11.3 MJ/kg and 17% crude protein on a DM basis (Keogh et al., 2023). Each day a weighed amount of the pelletized ration was provided to each pen in a self-feeder and any refusals from the previous day were collected and weighed. Lambs were weighed every 2 wk with the treatment mean LW used to calculate feeding levels for the subsequent 2 wk.

Feeding periods of 8 wk commenced when lambs were a mean age of 100 (±3.7 SD) and 230 (±3.7 SD) d, for the first and second feeding periods (P1 and P2), respectively. Treatments are referred to as H1 and L1 for P1 and H2 and L2 for P2. Half of the pens from each treatment in P1 were randomly selected and switched to the alternate treatment for P2, with remaining pens allocated to the same feeding-level treatment for both P1 and P2, thus creating 4 feeding treatment groups: HH, HL, LH, and LL. Lambs were managed on pasture paddocks prior to both feeding periods.

Lambs were weighed and CT scanned at the commencement and conclusion of both feeding periods. Twelve lambs (one pen from each of the 4 CT scanning timepoints) were randomly selected to assess the accuracy of estimating empty body composition using a reduced number of CT scan images taken at regular intervals throughout the body.

### CT scans

Lambs were scanned live and without sedation and were individually moved through the CT scanner (16 Slice Toshiba Alexion Advance) in a sternal recumbent position and secured by being strapped in a cradle. The CT scan image included legs in the scans but not the head. The X-ray tube energy setting was 120 kV. Cross-sectional slices (5 mm width) were taken between the 4th cervical vertebrae and the 1st sacral vertebrae, yielding an average of 120 images per animal. Slices of 5 mm width were chosen to provide sufficient detail to manually dissect empty body components whilst minimizing the time taken to CT scan the lambs. Images were manually edited (OsiriX https://www.osirix-viewer.com/, accessed 2021; Rosset et al., 2004) by a single operator by identifying and connecting lines along the boundaries of the reticulo-rumen, omasum, and abomasum (stomach) contents and viscera tissues to dissect the stomach and combined viscera tissues from the remainder of the body (Haynes et al., 2010; De Barbieri et al., 2015).

**Figure 1.**
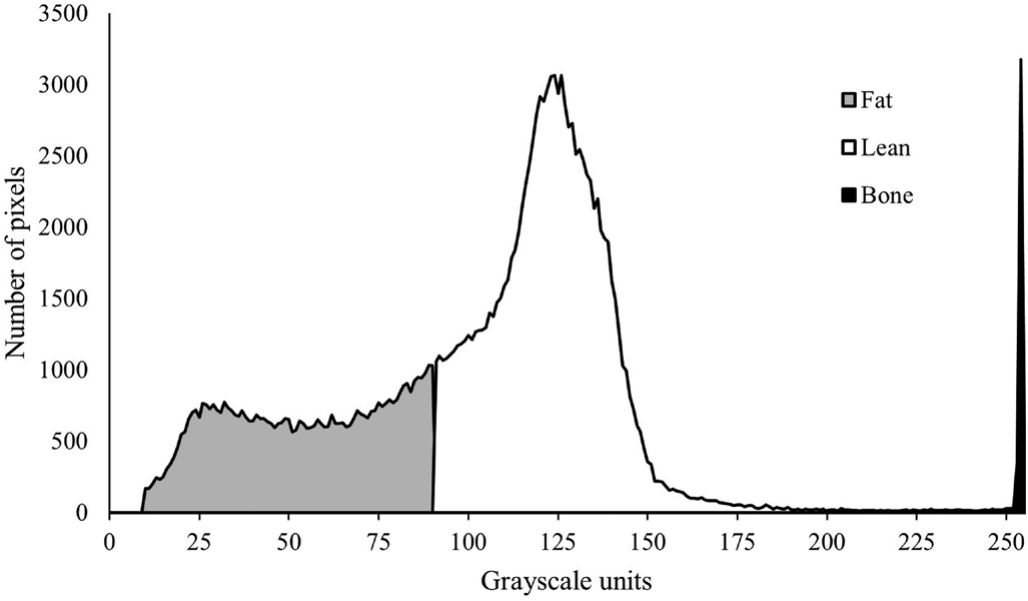
The number of pixels in each grayscale unit in a single 5 mm CT scan image of the forequarter of a lamb with pixels separated by the identified boundaries for fat, lean, and bone.

**Figure 2.**
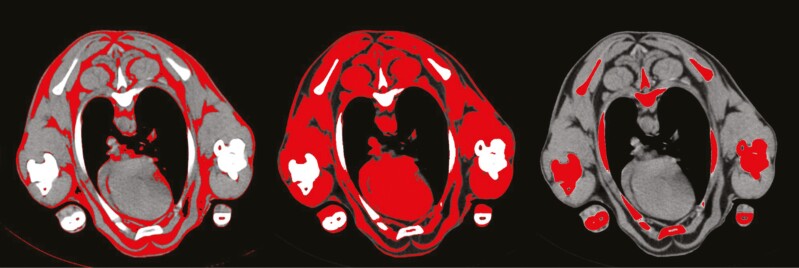
Three copies of a CT scan image of the forequarter of a lamb with fat, lean, and bone tissues highlighted (respectively from left to right) which were separated by grayscale unit boundaries. Boundaries for fat, lean, and bone were set to 10 to 90, 91 to 200, and 201 to 255, respectively.

The proportion of stomach-fill components (gas, particulate, and liquid) and the fat, lean, and bone composition of carcass, viscera, and the empty body (combined carcass and viscera with stomach removed) were then determined using Image J (v1.53, https://imagej.net/ij/, accessed 2021) to create a distribution of pixels in grayscale units from 0 to 255 (Figure 1). Component boundaries were identified in Image J by observing highlighted components at different grayscale thresholds (Figure 2) in slices taken at 30 mm intervals from 10 randomly selected lambs at each CT scanning timepoint (40 lambs total). Boundaries for fat, lean, and bone were set to 10 to 90, 91 to 200, and 201 to 255, respectively, which correspond well with the boundaries outlined by Alston et al. (2004) and Kvame and Vangen (2006). For the stomach components, boundaries for gas, particulate, and liquid were set to 0 to 1, 2 to 65, and 65 to 255, respectively.

All CT scan images (5 mm intervals) taken throughout the body from 3 randomly selected lambs at each of the 4 CT scanning timepoints were used to determine the accuracy of using a reduced number of images at various regular intervals for estimating the mass and volume of empty body components. The use of representative scans from each of the 3 body regions (forequarter, mid-section, and hindquarter) similar to the method used by Kvane and Vangen (2006) and Macfarlane et al. (2006) are also reported; 3 representative scans from the forequarter (4^th^ cervical to 6^th^ thoracic vertebrae) and hindquarter (5^th^ lumbar to 4^th^ sacral vertebrae) were taken at 50 mm intervals and in the mid-section (6^th^ thoracic to 5^th^ lumbar vertebrae) at 100 mm intervals. The first representative scan site for each section was the most cranial image available within the range of anatomical landmarks described above.

### Calculations

The proportion of components in the empty body, carcass, viscera, and reticulo-rumen, omasum, and abomasum (stomach) was calculated by allocating pixels based on the grayscale boundaries described above. The total number of pixels within the boundaries for each tissue type was summed for each lamb using all available images for the stomach, viscera, and empty body (at 15 mm intervals for the stomach and 30 mm intervals for remainder of the empty body). The number of pixels for each tissue type (e.g., bone, lean, and fat) within each component (stomach, viscera, and empty body) was then used to calculate the area occupied by each tissue by dividing by the sum of pixels for all tissues types present in the available images.

The calculated proportions of each tissue type were corrected for tissue density to determine tissue mass (Fullerton, 1980). Tissue densities of 0.95, 1.05, and 1.45 g/cm^3^ were used for fat, lean, and bone, respectively (Fullerton, 1980; Young et al., 1996; Campbell et al., 2003). For the stomach contents, densities of 0, 0.65, and 1 g/cm^3^ were used for gas, particulate, and liquid, respectively (Bain et al., 2014).

The stomach and viscera volumes were calculated within the computer program OsiriX with CT images at 15- and 30-mm intervals, respectively. Stomach contents and viscera mass were estimated by multiplying the calculated volume by the proportions of stomach-fill components (gas, particulate, and liquid), and fat and lean tissue, respectively, which were corrected for tissue density (Equations 1 to 4).


$$\begin{array}{l} Stomach\;component=stomach\;volume\times proportion\times density \end{array}$$ (1)

$$\begin{array}{l} Stomach\;contents=gas+particulate+liquid \end{array}$$ (2)

$$\begin{array}{l} Viscera\;component=(viscera\;volume-stomach\;volume)\times proportion\times density \end{array}$$ (3)

$$\begin{array}{l} Viscera=viscera\;lean+viscera\;fat \end{array}$$ (4)

Calculations of empty body tissue mass required a combination of LW, CT scan data, an estimation of fleece weight, an estimation of the weight of the head and feet, and an estimation of the weight of contents of the stomach to correct for gut fill.

Fleece-free empty body weight (**FFEBW**) was estimated from LW minus the estimated weight of stomach contents and estimated weight of fleece (Equation 5). Wool growth (clean fleece/d) was estimated using the model described by Oddy et al. (2024) with a breed factor value of 0.6 and calculations were divided by 0.7 to allow for the approximate ratio of greasy fleece weight to clean fleece weight. The fleece was estimated to weigh 1.0 kg at the commencement of both feeding periods due to the lambs being shorn shortly after the conclusion of the first feeding period.


$$\begin{array}{l} FFEBW=LW-stomach\;contents-fleece \end{array}$$ (5)

$$\begin{array}{l} Head\;and\;feet=0.05\times FFEBW+1.5 \end{array}$$ (6)

$$\begin{array}{l} Carcass\;component=(FFEBW-head\;and\;feet-viscera)\times proportion\times density \end{array}$$ (7)

$$\begin{array}{l} NVEB=Carcass\;lean+head\;and\;feet+bone \end{array}$$ (8)

$$\begin{array}{l} Lean\;tissue=NVEB+viscera\;lean \end{array}$$ (9)

For calculations of fat-free non-viscera empty body (**NVEB**) tissue weight; the approximate weight of the head and feet was calculated using Equation 6 which was developed from data reported by Hegarty et al. (1999) and Dougherty et al. (2022). The estimated weight of visceral tissues was subtracted from FFEBW minus the weight of the head and feet, and this value was used to calculate carcass tissue weights using the fat, lean, and bone tissue proportions from CT scan image analysis (Equation 7). The approximate weight of the head and feet, and bone was added to carcass lean tissue weight to calculate NVEB mass (Equation 8). To compare predictions of total lean tissue mass using predictive growth models to CT-derived values, visceral lean tissue mass was added to NVEB mass to estimate total lean mass (Equation 9).

### Model description

Equations for the CSIRO and Oddy models were computed using Excel (Microsoft, 2019) to predict FFEBW, lean, and fat tissue mass at the conclusion of each period for H and L feeding-level treatments. The Oddy model additionally separates lean tissue into NVEB and viscera components which are presented here both separately and combined as lean tissue for comparisons with the CSIRO model. A standard reference weight (**SRW**) of 70 kg was used for both models which was multiplied by 0.92 to account for approximate gut fill (CSIRO, 2007) so that the proportion of initial FFEBW to adjusted SRW could be used to determine stage of maturity.

The CT scan-derived FFEBW and empty body tissue mass, referred to as CT or observed values (i.e., FFEBW, fat, lean, viscera lean, and NVEB), were calculated on a pen basis (3 lambs per pen) as individual animal energy intake was unknown. The models were run separately for each pen using the initial values for body composition as determined by CT scans. Metabolizable energy intake was averaged over the feeding periods.

In the CSIRO model, the energy value of protein including wool (23.6 kJ/g) and fat (39.3 kJ/g) was as described by CSIRO (2007) and protein was determined to be 21.6% of fat-free mass. The Oddy model used similar values for protein (23.8 kJ/g) and fat (39.6 kJ/g) and protein was determined to be 21% of fat-free mass in the NVEB and 15.7% of fat-free mass in the viscera (Dougherty et al., 2020*a*, 2020*b*).

It was necessary that the same fleece growth model be utilized by both models as wool growth is a source of retained energy and any discrepancy in calculating FFEBW would have also affected predicted empty body tissue mass.

### Statistical analysis

Root mean square prediction errors (**RMSPE**) were calculated in Excel (Microsoft, 2019) for the empty body component volume and tissue mass estimated using a reduced number of CT scan slices at varying intervals in comparison to using all available scans throughout the body in 12 lambs. Regression coefficients were analyzed by generalized linear models in RStudio (RStudio Team, 2020) with fixed effects of treatment, sex, sire, CT scan date, and LW.

The CT scan data from individual lambs were analyzed by generalized linear models using ASReml (Gilmour et al., 2009). Lamb LW and empty body composition were modeled using a linear univariate model with fixed effects of treatment, sire, sex, CT scan date, birthweight, age, LW at the commencement of P1, the interaction of P1 and P2 treatment for the analysis of P2 results and pen as a random effect.

A comparison of model-predicted FFEBW, empty body tissue mass, and CT values was evaluated in RStudio (RStudio Team 2020). The coefficient of variation of CT values and mean bias of the predicted means are reported. Mean bias was calculated as the sum of the CT value minus the predicted value for all pens by treatment within feeding period, divided by the number of pens.

Mean square prediction error (**MSPE**) was calculated as the sum of the CT value minus the model-predicted value squared for all pens by treatment within feeding period, divided by the number of pens. The RMSPE was the square root of the MSPE and is reported as a percentage of the CT value. Mean bias and RMSPE were used to compare predicted values as they are in the same unit as the CT value.

The MSPE was decomposed into mean bias, slope bias, and random error as described by Tedeschi (2006), Ellis et al. (2009), and Oddy et al. (2024). The mean bias calculated is the bias of the prediction and is zero when the mean predicted value coincides with the mean CT value. The slope bias calculated the error due to deviation of the regression slope from unity and the random error calculated the error due to disturbance, i.e., the portion of the MSPE that cannot be eliminated by linear corrections of the predictions. Random error largely reflects variation due to measurement errors.

## Results

### Development of CT scan analysis method using 12 lambs

RMSPE is presented for the estimated empty body composition, estimated stomach, and viscera volume, and the proportions of stomach-fill components using 12 lambs with varying slice intervals (Table 1). RMSPE increased for all tissues with increasing slice intervals with more rapid increases seen in tissues with smaller mean tissue mass (i.e., bone and viscera fat). All components had RMSPE less than 1% of the mean for slice intervals 30 mm or less except for bone and viscera fat. The use of representative scans had similar accuracies to using CT scan images at 60 mm intervals throughout the empty body and carcass.

**Table 1.** RMSPE for estimated empty body composition and stomach and viscera volumes using CT scans of lambs (*n* = 12) with varying slice intervals

	Mean mass/volume^1^ (±SE)	RMSPE (%)					
Slice interval		10 mm	15 mm	30 mm	45 mm	60 mm	3 scans/region^2^
Empty body							
Fat, kg	7.0 ± 0.82	0.04	0.11	0.11	0.29	0.46	0.49
Lean, kg	19.7 ± 0.63	0.01	0.01	0.03	0.04	0.10	0.07
Bone, kg	3.7 ± 0.08	0.06	0.17	0.49	1.13	1.59	1.72
Carcass + skin							
Fat, kg	5.0 ± 0.59	0.07	0.19	0.26	0.48	0.74	0.60
Lean, kg	14.4 ± 0.41	0.01	0.02	0.04	0.08	0.12	0.13
Bone, kg	3.7 ± 0.09	0.08	0.13	0.45	1.07	1.51	1.71
Viscera							
Fat, kg	2.0 ± 0.37	0.28	0.40	0.38	1.48	2.93	3.98
Lean, kg	5.2 ± 0.61	0.02	0.07	0.13	0.27	0.44	1.11
Volume, L	15.4 ± 0.67	0.33	0.46	1.04	1.44	—	—
Stomach contents							
Particulate, kg	0.6 ± 0.29	9.40	17.07	22.49	35.66	—	—
Liquid, kg	4.3 ± 0.25	0.03	0.05	0.07	0.14	—	—
Total mass, kg	4.9 ± 0.28	0.25	0.42	0.76	1.45	—	—
Volume, L	5.5 ± 0.40	1.22	1.61	2.62^3^	3.38^4^	—	—

^1^Mean mass predicted using animal LW and CT scan slices at intervals of 5 mm throughout the body.

^2^Three CT scan slices taken from forequarter, mid-section, and hindquarter. Similar to CT scan analysis method reported by Kvane and Vangen (2006) and Macfarlane et al. (2006).

^3^Slice interval 20 mm.

^4^Slice interval 25 mm.

The following regression coefficients compare alternate analysis methods to estimates determined by CT scan slices of 5 mm thickness taken throughout the entire body. When analyzed using 3 representative scans from each body region, similar to the method used by Kvane and Vangen (2006) and Macfarlane et al. (2006), the *R*^2^ was 0.97, 0.94, and 0.83 for carcass fat, lean, and bone, respectively, and 0.95 and 0.96 for viscera fat and lean, respectively. When analyzed using 30 mm intervals between CT scan slices, the *R*^2^ was 0.99, 0.99, and 0.92 for carcass fat, lean, and bone, respectively, and 0.99 for both viscera fat and lean.

The mean stomach volume ± SE was 5.5 ± 0.4 L and the mean viscera volume ± SE was 15.4 ± 0.7 L, which includes viscera lean and fat, stomach contents, and any gas in the abdomen or thoracic cavity. The differences in analyzed slice interval between the stomach and viscera are due to the smaller volume of the stomach and decreased number of involved slices. Stomach volume RMSPE increased from 1.2% to 3.4% by increasing slice interval from 10 to 25 mm in comparison to viscera volume which increased from 0.3% to 1.4% by increasing slice interval from 10 to 45 mm.

RMSPE increased for stomach-fill components with increasing slice interval and greater increases were seen in particulate which had a smaller mean mass. Particulate RMSPE increased from 9.4% to 35.7%, whereas liquid RMSPE increased from 0.03% to 0.14% by increasing slice interval from 10 to 45 mm.

### Calculation of empty body composition

LW, estimated fleece weight, stomach contents mass, FFEBW, weight of the head and feet, and CT mass of empty body tissues are presented as raw unadjusted means (±SE) in Table 2. There were no significant interactions between diet treatment and feeding period and the treatments within each period are, therefore, presented separately.

**Table 2.** Effect of feeding level on lamb tissue weights (*n* = 108) at the beginning and end of each feeding period (mean ± SE)

	Beginning		End	
	Low^1^	High^2^	Low^1^	High^2^
Feeding period 1				
LW, kg	31.2 ± 0.46	32.3 ± 0.38	36.0 ± 0.68^a^	44.4 ± 0.52^b^
Fleece weight^3^, kg	1.0	1.0	1.3 ± 0.00	1.4 ± 0.00
Rumen volume, L	4.9 ± 0.17	5.1 ± 0.13	5.3 ± 0.20	5.6 ± 0.17
Rumen contents, kg	4.1 ± 0.15	4.3 ± 0.12	4.5 ± 0.19	5.0 ± 0.15
FFEBW^4^, kg	26.1 ± 0.38	27.0 ± 0.32	30.4 ± 0.56^a^	38.0 ± 0.47^b^
Head and feet, kg	2.8 ± 0.02	2.9 ± 0.02	3.0 ± 0.03	3.4 ± 0.02
Fat tissue, kg	4.1 ± 0.14	4.3 ± 0.13	6.0 ± 0.21^a^	9.1 ± 0.21^b^
Bone, kg	3.0 ± 0.05	3.1 ± 0.05	3.5 ± 0.05^a^	3.9 ± 0.06^b^
Viscera lean tissue, kg	5.1 ± 0.10	5.1 ± 0.10	4.0 ± 0.08^a^	5.4 ± 0.10^b^
NVEB^5^ mass, kg	17.0 ± 0.25	17.6 ± 0.24	20.4 ± 0.34^a^	23.6 ± 0.30^b^
Lean tissue^6^, kg	22.0 ± 0.31	22.7 ± 0.29	24.4 ± 0.40^a^	28.9 ± 0.37^b^
Feeding period 2				
LW, kg	40.8 ± 0.51	40.5 ± 0.53	45.0 ± 0.55^a^	51.4 ± 0.53^b^
Fleece weight^4^, kg	1.0	1.0	1.4 ± 0.00	1.5 ± 0.01
Rumen volume, L	7.6 ± 0.21	7.5 ± 0.21	6.1 ± 0.15	6.0 ± 0.12
Rumen contents, kg	6.8 ± 0.22	6.6 ± 0.20	5.3 ± 0.14	5.3 ± 0.12
FFEBW^4^, kg	33.0 ± 0.38	33.0 ± 0.47	38.3 ± 0.48^a^	44.6 ± 0.48^b^
Head and feet, kg	3.2 ± 0.02	3.1 ± 0.02	3.4 ± 0.02	3.7 ± 0.02
Fat tissue, kg	5.6 ± 0.20	5.6 ± 0.20	9.2 ± 0.24^a^	12.4 ± 0.23^b^
Bone, kg	3.7 ± 0.05	3.7 ± 0.06	4.2 ± 0.06	4.3 ± 0.06
Viscera lean tissue, kg	5.5 ± 0.08	5.5 ± 0.12	4.7 ± 0.08^a^	5.7 ± 0.09^b^
NVEB^5^ mass, kg	21.9 ± 0.26	21.8 ± 0.31	24.4 ± 0.31^a^	26.5 ± 0.33^b^
Lean tissue^6^, kg	27.4 ± 0.30	27.3 ± 0.40	29.1 ± 0.35^a^	32.2 ± 0.40^b^

^1^Low level of feeding (DM intake 2.5% and 2.4% of LW for periods 1 and 2, respectively).

^2^High level of feeding (DM intake 3.6% and 3.3% of LW for periods 1 and 2, respectively).

^3^Fleece weight was assumed to weigh 1.0 kg at the beginning of both feeding periods due to the animals being shorn between the feeding periods and values at the end were predicted as described by Oddy et al. (2024).

^4^Fleece free empty body weight.

^5^Fat-free NVEB estimated using Equation 8.

^6^Lean is defined as sum of fat-free empty body mass estimated using Equation 9.

^a,b^Unlike superscripts at the end of each feeding period differ (*P* < 0.05). Data shown are unadjusted means reported by feeding period and treatment.

Initial values did not differ (*P* > 0.05) between treatment groups.

At the commencement of P1, there were no significant differences between the mass of components between P1 treatments and lambs had a mean LW of 31.8 ± 0.4 kg and mean FFEBW of 26.6 ± 0.3 kg. At the conclusion of P1, lambs in the L1 treatment were lighter (*P* < 0.001) with a mean LW of 36.0 ± 0.7 kg and mean FFEBW of 30.4 ± 0.6 kg compared with H1 lambs with a mean LW of 44.4 ± 0.5 kg and mean FFEBW of 38.0 ± 0.5 kg. Lambs in the L1 treatment also contained less fat (6.0 ± 0.2 vs. 9.1 ± 0.2 kg; *P* < 0.001), lean (24.4 ± 0.4 vs. 28.9 ± 0.4 kg; *P* < 0.001), viscera lean (4.0 ± 0.1 vs. 5.4 ± 0.1 kg; *P* < 0.001) and bone (3.5 ± 0.1 vs. 3.9 ± 0.1 kg; *P* < 0.001) in the empty body than H1 lambs.

At the commencement of P2, there were no differences between P2 treatments due to the reallocation of half of the lambs to different treatments from P1 to P2 and lambs had a mean LW of 40.7 ± 0.5 kg and mean FFEBW of 33.0 ± 0.4 kg. At the conclusion of P2, lambs in the L2 treatment were lighter (*P* < 0.001) with a mean LW of 45.0 ± 0.6 kg and mean FFEBW of 38.3 ± 0.5 kg compared with H2 lambs with a mean LW of 51.4 ± 0.5 kg and mean FFEBW of 44.6 ± 0.5 kg. Lambs in the L2 treatment also contained less fat (9.2 ± 0.2 vs. 12.4 ± 0.2 kg; *P* < 0.001), lean (29.1 ± 0.4 vs. 32.2 ± 0.4 kg; *P* < 0.001) and viscera lean (4.7 ± 0.1 vs. 5.7 ± 0.1 kg; *P* < 0.001) with no differences in bone (4.2 ± 0.1 vs. 4.3 ± 0.1 kg).

**Figure 3.**
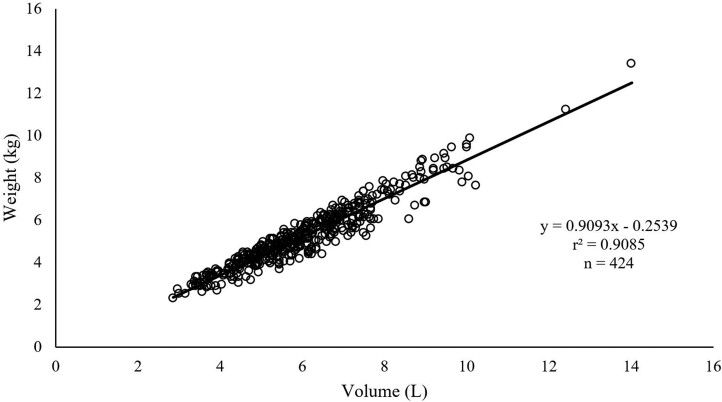
Relationship between volume and weight of reticulo-rumen, omasum, and abomasum estimated by CT scanning.

The volume and weight of the stomach contents estimated from CT scanning were highly correlated (*R*^2^ = 0.91) and were unaffected by treatments at each scanning time point (Figure 3).

### Comparison of observed empty body composition with model predictions

The data for the following comparison is shown in Table 3 which indicates that overall, the CSIRO model does better at predicting fat mass and FFEBW than the Oddy model, and predictions of lean mass by both models are similar. Mean bias and RMSPE were the largest for fat tissue mass, particularly in P2 using both predictive models. Predicted fat tissue mass was greater than observed fat for P1 lambs and lower than observed fat for P2 lambs using the CSIRO model. The predicted fat tissue mass by the CSIRO model showed the largest mean bias (1.6 kg) and RMSPE (13.4%) for the H2 lambs. Over 50% of the error for the prediction of fat tissue mass using the CSIRO model was attributed to random error in P1 and mean bias in P2.

**Table 3.** Comparison of the CSIRO (2007) model and Oddy et al. (2024) model for the prediction of lamb FFEBW and body composition at the end of each feeding period (*n* = 108)

	Feeding period 1				Feeding period 2			
	Low^1^		High^2^		Low^1^		High^2^	
Fat (kg)								
Observed mean^3^	6.0		9.1		9.2		12.4	
CV of observations (%)	14.1		10.8		13.2		8.5	
	Oddy	CSIRO	Oddy	CSIRO	Oddy	CSIRO	Oddy	CSIRO
Mean of predictions	5.5	6.3	8.2	9.4	7.4	8.5	9.6	10.8
Mean bias	0.5	−0.3	0.9	−0.3	1.8	0.7	2.8	1.6
RMSPE^4^ (% observed mean)	11.7	8.9	10.9	6.4	20.1	8.6	23.1	13.4
Error decomposition (% MSPE^5^)								
Mean bias	58.1	24.6	76.6	22.7	94.8	69.9	98.1	94.0
Slope bias	0.9	2.2	1.7	4.4	0.0	0.2	5.5	0.1
Random error	40.9	73.3	21.7	72.9	5.2	29.9	1.9	6.0
Lean^6^ (kg)								
Observed mean^3^	24.6		28.9		29.1		32.3	
CV of observations (%)	6.9		5.8		6.0		7.1	
	Oddy	CSIRO	Oddy	CSIRO	Oddy	CSIRO	Oddy	CSIRO
Mean of predictions	24.3	25.3	28.4	28.5	29.7	30.2	32.2	31.8
Mean bias	0.3	−0.6	0.5	0.4	−0.6	−1.1	0.1	0.4
RMSPE^4^ (% observed mean)	4.3	5.0	4.3	3.9	3.8	4.8	3.5	3.0
Error decomposition (% MSPE^5^)								
Mean bias	10.6	26.1	15.4	13.4	29.6	60.4	0.2	18.7
Slope bias	0.1	0.9	0.1	4.1	18.3	7.0	40.3	4.1
Random error	89.3	73.0	84.5	87.7	52.1	32.6	59.5	77.2
**FFEBW (kg)**								
Observed mean^3^	30.7		38.0		38.3		44.7	
CV of observations (%)	6.7		4.2		5.9		5.0	
	Oddy	CSIRO	Oddy	CSIRO	Oddy	CSIRO	Oddy	CSIRO
Mean of predictions	29.8	31.5	36.7	37.9	37.1	38.7	41.8	42.6
Mean bias	0.9	−0.9	1.4	0.1	1.2	−0.4	2.9	2.0
RMSPE^4^ (% observed mean)	5.1	5.2	4.7	2.9	4.1	2.7	7.0	5.0
Error decomposition (% MSPE^5^)								
Mean bias	32.5	30.8	57.5	1.4	58.1	16.1	87.0	81.6
Slope bias	0.0	0.8	1.4	4.0	4.9	26.4	0.6	0.1
Random error	67.5	68.4	41.1	94.7	37.1	57.4	12.5	18.3

^1^Low level of feeding (DMI 2.5% and 2.4% of LW for periods 1 and 2, respectively).

^2^High level of feeding (DMI 3.6% and 3.3% of LW for periods 1 and 2, respectively).

^3^Observed mean refers to data as measured using CT scans. Data shown are unadjusted means reported by feeding period and treatment.

^4^Root mean square prediction error.

^5^Mean square prediction error and its decomposition, as per Tedeschi (2006), Ellis et al. (2009), and Oddy et al. (2024).

^6^Lean is defined as sum of fat-free empty body mass estimated using Equation 9.

Predicted fat tissue mass was lower than observed fat predicted by the Oddy model for all treatments and to the greatest extent for P2 lambs. The predicted fat tissue mass by the Oddy model showed the largest mean bias (2.8 kg) and RMSPE (23.1%) for the H2 lambs. Over 50% of the prediction error using the Oddy model was attributed to mean bias for all treatments.

**Figure 4.**
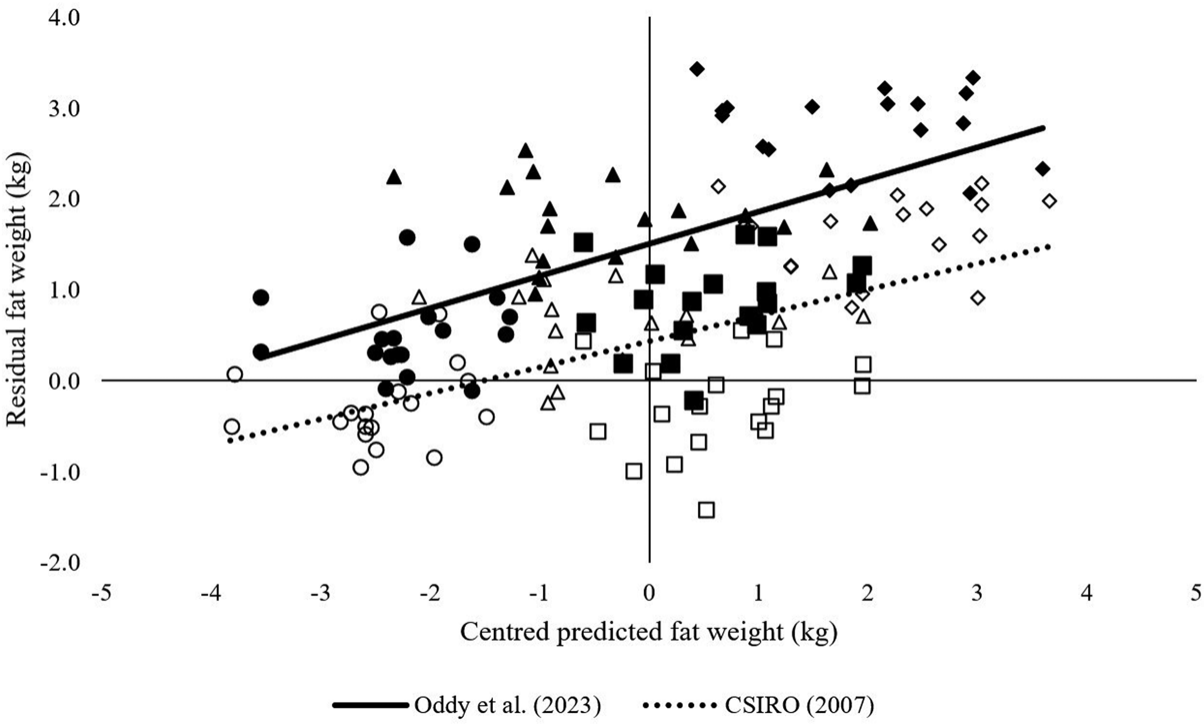
Predicted fat weight centered around the mean and the residuals of predicted values calculated using models described by CSIRO (2007; hollow symbols) and Oddy et al. (2024; solid symbols) at the conclusion of both feeding periods. Symbol shapes illustrate low (circle) and high (square) feeding level in period 1 and low (triangle) and high (diamond) feeding level in period 2.

The overall mean predicted fat tissue mass across all treatments using the Oddy model had a mean bias of 1.5 kg whereas the CSIRO model had a mean bias of 0.4 kg (Figure 4). For predicted fat tissue mass across all treatments, 69.6% of the error was attributed to mean bias using the Oddy model and 18% random error, whereas 18.6% of the error was attributed to mean bias using the CSIRO model and 54% attributed to random error. The slope of the trendlines was similar irrespective of the model used.

Observed lean tissue mass (sum of NVEB and viscera lean) was closely aligned with model-predicted values with a RMSPE of 5% or less for all treatments and using both models. The greatest mean bias and RMSPE occurred using the CSIRO model to predict the L treatments with over 50% of the error attributed to mean bias for L2. Random error contributed greater than 50% to all other treatments using both models.

**Figure 5.**
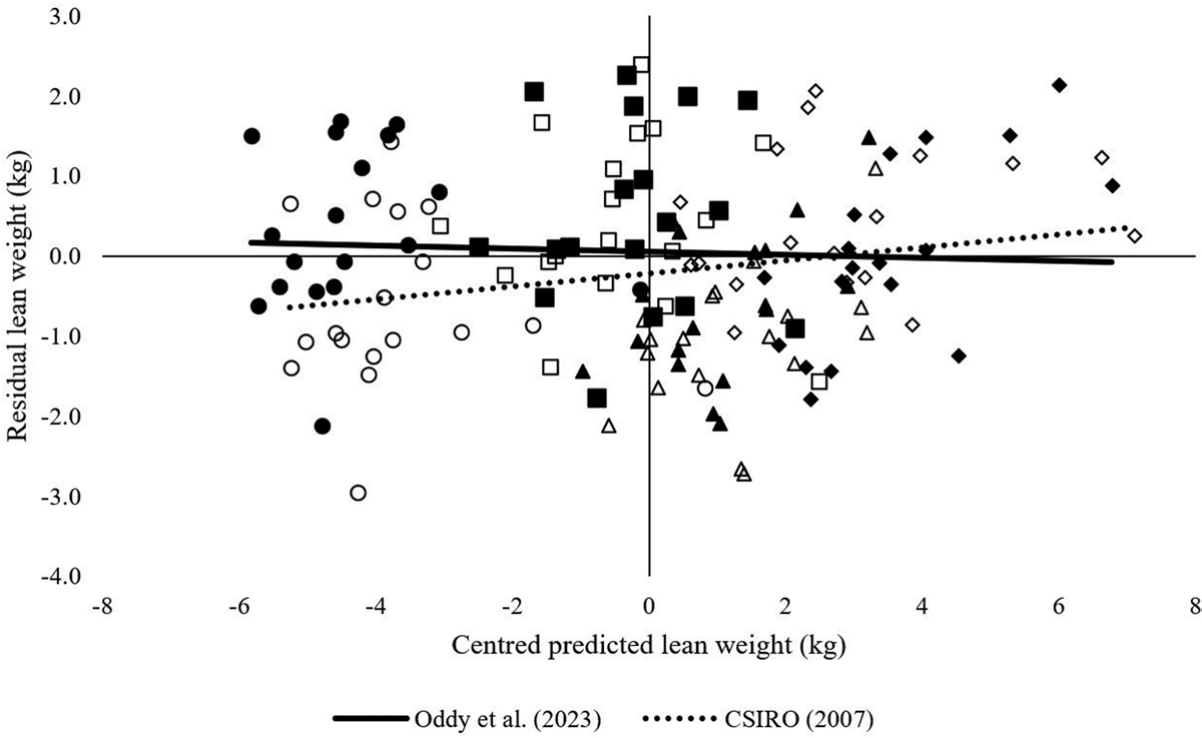
Predicted lean weight (NVEB and viscera lean) centered around the mean and the residuals of predicted values calculated by models described by CSIRO (2007; hollow symbols) and Oddy et al. (2024; solid symbols) at the conclusion of both feeding periods. Symbol shapes illustrate low (circle) and high (square) feeding level in period 1 and low (triangle) and high (diamond) feeding level in period 2.

The overall mean predicted lean tissue mass across all treatments using the Oddy model had a mean bias of 0.1 kg whereas the CSIRO model had a mean bias of −0.2 kg (Figure 5). For predicted lean tissue mass, 0.4% of the error was attributed to mean bias using the Oddy model and 99.5% random error whereas 3.4% of the error was attributed to mean bias using the CSIRO model and 92.9% attributed to random error.

The prediction of FFEBW showed the largest discrepancy with observed FFEBW in H2 lambs using both models and both demonstrated over 80% of the prediction error was attributed to mean bias. Predictions of FFEBW were greater than observed for L treatments and lower than observed for H treatments using the CSIRO model. The greatest mean bias (2.0 kg) and RMSPE (5.0%) were for the H2 lambs. Over 50% of the prediction error using the CSIRO model was attributed to random error for all treatments except H2 with 81.6% attributed to mean bias. Predictions of FFEBW were lower than observed for all treatments using the Oddy model. The greatest mean bias (2.9 kg) and RMSPE (7.0%) were for the H2 lambs with over 50% of the prediction error attributed to mean bias for all treatments except L1 with 67.5% attributed to random error.

### Comparison of NVEB and viscera lean with model predictions

**Figure 6.**
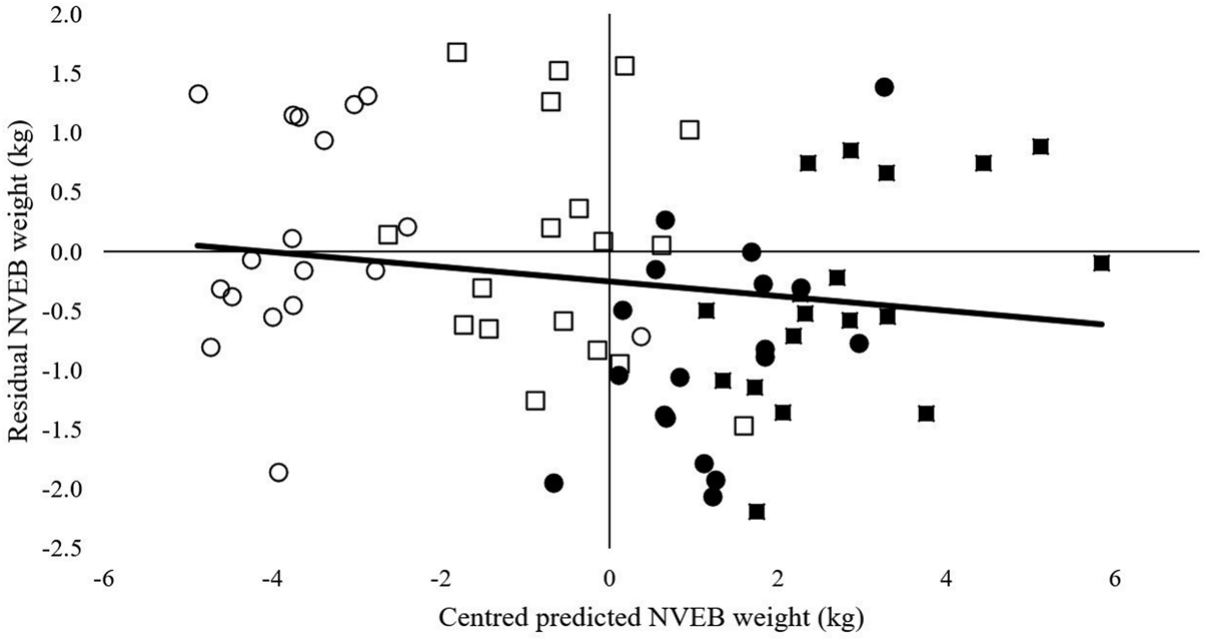
Predicted fat-free NVEB mass centered around the mean and the residuals of predicted values calculated by the model described by Oddy et al. (2024) at the conclusion of both feeding periods. Symbol shapes illustrate low (circle) and high (square) feeding level in period 1 (hollow symbols) period 2 (solid symbols).

The prediction of NVEB tissue by the Oddy model closely matched the observed NVEB with over 98% of the error attributed to random error in P1 (Table 4). In P2, over 50% of the error was associated with random error and slope bias for both feeding-level treatments. The mean predicted NVEB tissue mass across all treatments using the Oddy model had a mean bias of −0.2 kg with 92.2 % of the error attributed to random error (Figure 6).

**Table 4.** Comparison of the Oddy et al. (2024) model for the prediction of lamb fat-free NVEB and viscera lean mass at the end of each feeding period (*n* = 108)

	Feeding period 1		Feeding period 2	
	Low^1^	High^2^	Low^1^	High^2^
NVEB (kg)				
Observed mean^3^	20.6	23.6	24.4	26.5
CV of observations (%)	7.2	5.8	6.7	7.1
Mean of predictions	20.5	23.5	25.3	26.9
Mean bias^1^	0.1	0.1	−0.8	−0.3
RMSPE^4^ (% CT scan mean)	4.3	4.3	4.9	3.6
Error decomposition (% MSPE^5^)				
Mean bias	1.5	0.6	46.7	11.6
Slope bias	7.5	1.2	13.0	20.0
Random error	98.4	98.2	40.3	68.5
Viscera lean (kg)				
Observed mean^3^	4.0	5.4	4.7	5.7
CV of observations (%)	7.0	8.5	6.1	8.6
Mean of predictions	3.8	4.9	4.5	5.3
Mean bias	0.2	0.4	0.2	0.4
RMSPE^4^ (% CT scan mean)	8.0	10.2	7.1	9.5
Error decomposition (% MSPE^5^)				
Mean bias	53.9	58.4	41.2	48.1
Slope bias	5.3	16.6	2.6	32.4
Random error	40.8	25.0	56.3	19.5

^1^Low level of feeding (DM intake 2.5% and 2.4% of LW for periods 1 and 2, respectively).

^2^High level of feeding (DM intake 3.6% and 3.3% of LW for periods 1 and 2, respectively).

^3^Observed mean refers to data as measured using CT scans. Data shown are unadjusted means reported by feeding period and treatment.

^4^Root mean square prediction error.

^5^Mean square prediction error and its decomposition, as per Tedeschi (2006), Ellis et al. (2009), and Oddy et al. (2024).

**Figure 7.**
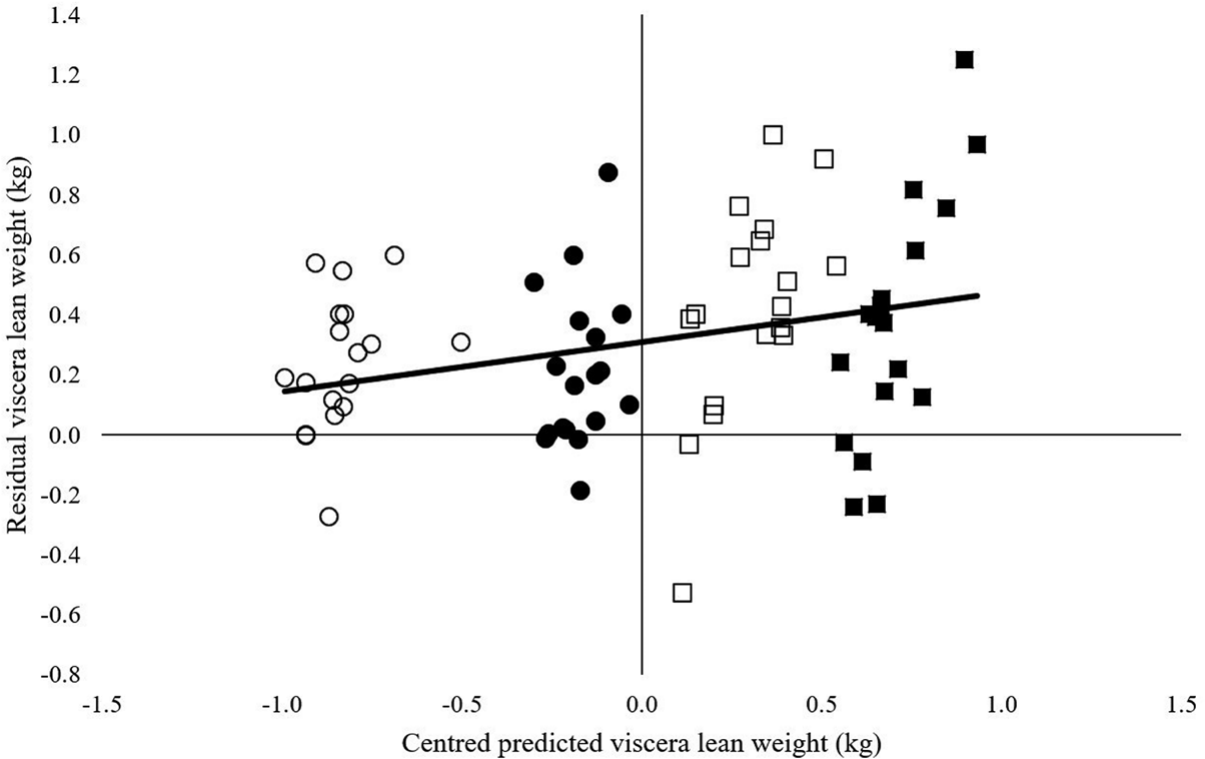
Predicted viscera lean weight centered around the mean and the residuals of predicted values calculated by the model described by Oddy et al. (2024) at the conclusion of both feeding periods. Symbol shapes illustrate low (circle) and high (square) feeding level in period 1 (hollow symbols) period 2 (solid symbols).

Predicted viscera lean tissue was lower than observed viscera lean for all treatments with a mean bias of 0.4 kg for H treatments and 0.2 kg for L treatments. Greater than 50% of the error was attributed to mean bias for P1 treatments and greater than 40% attributed to mean bias for P2 treatments. Across all treatments, the mean predicted viscera lean tissue mass had a mean bias of 0.3 kg with 47.9% of the error attributed to mean bias and 47.3% random error (Figure 7).

## Discussion

The current study demonstrates the capability of CT scanning of live lambs to estimate changes in body composition at different stages of maturity and in response to changing feeding levels. Although some precision may be lost by using a limited number of CT scan slices, whether by increasing slice interval or by using representative scans, the output is highly correlated with data obtained from using all images throughout the body, see also Kvane and Vangen (2006) and Macfarlane et al. (2006). CT scans remove several limitations of balance and serial slaughter studies. Most notably, the use of CT scanning of live lambs means a greater amount of data can be generated whilst reducing the number of animals involved in experiments as changes can be measured at multiple timepoints. To replicate the same data from a comparative slaughter study it would require 4 times the animals, as each individual was CT scanned 4 times, and additional laboratory work would be required for analysis. More importantly, it allows serial measurement of the same animal reducing error due to differences between animals.

The lean tissue mass predicted by the CSIRO and Oddy models was similar to CT lean tissue mass, demonstrating the ability of CT scans to inform predictive growth models of changes in body composition during lamb growth. Models based on body compositional data obtained from different serial slaughter experiments closely match the measured values, suggesting CT scans have utility. Other researchers have demonstrated the high accuracy of CT scan-derived estimates of body composition when compared with comparative slaughter measurements (Macfarlane et al., 2006; De Barbieri et al., 2015). The comparison of CT scans with model predictions highlighted that either both models are susceptible to errors in the prediction of fat mass, especially at later stages of maturity, or that the method used to calculate CT fat tissue mass was erroneous.

The CSIRO model is limited by its inability to adapt to changing visceral lean mass following a change in feeding levels or diet composition. Additionally, the CSIRO model uses a single term for the efficiency of energy utilization for gain, irrespective of feeding level and stage of maturity, which has been shown to be problematic (Graham, 1980; Keogh et al., 2023). The Oddy model better represents the realities of energy transactions in the growing ruminant and with further refinement, it is likely to be a valuable tool. The Oddy model underpredicted fat mass to a greater extent, and a greater proportion of the prediction error was attributed to mean bias suggesting that heat production was overestimated or the factor converting fat energy to weight was incorrect. The comparison of CT scan data with the Oddy model highlighted specific areas for further investigation to advance our understanding of ruminant energy transactions and the tissue responses to a changing feed supply.

### Prediction of empty body composition

The proportion of fat, lean, and bone can be accurately predicted from the use of 2 to 3 CT scan slices at 3 anatomical landmarks throughout the body (Kvane and Vangen, 2006). Whilst the current study and that of Kvane and Vangen (2006) did not verify results with slaughter data, previous researchers have demonstrated high levels of accuracy between CT-derived estimates of carcass composition and the dissection of slaughtered lambs (Sehested, 1984; Vangen and Jopson, 1996; Macfarlane et al., 2006; Haynes et al., 2010). Kvane and Vangen (2006) also identified that the main source of error was associated with predicting the weight of bone which has the smallest mass and demonstrates the greatest variation in mass throughout the body.

Lambs were CT scanned in an upright position with their legs underneath them in the current study which differed to that of previous researchers (Kvane and Vangen, 2006; Macfarlane et al., 2006). This made the selection of similar anatomical sites to those of previous researchers only possible for the mid-section of the animals. Regardless, the use of a similar approach using representative scans from each region produced almost identical correlations to those of Kvane and Vangen (2006) and Macfarlane et al. (2006) for the prediction of carcass composition. The alternative approach for the analysis of body composition is the *Cavalieri method* in which cross-sectional slices are taken at evenly spaced intervals along the entire body. This method shows similar accuracies in sheep for carcass analysis although a greater number of images are required to be manually edited (Bunger et al., 2011). The *Cavalieri method* has the added benefit of the prediction of volume of components by multiplying the isolated area by the distance between slices (Ball et al., 1997; Bunger et al., 2011).

In the current study, CT scan estimated viscera fat showed the greatest increase in RMSPE with a decreasing number of CT scan slices which corresponds with the findings of Thompson and Kinghorn (1992). For the accurate separation of body fat into carcass and non-carcass depots, at least 10 to 15 scans of the abdominal cavity were required, generally making a total of 20 to 25 scans for the whole animal. Due to the necessity to estimate both stomach and viscera volumes and viscera composition, it was determined that the *Cavalieri method* would be most appropriate for the current analysis. Irrespective of this, reference scans showed high levels of accuracy in determining empty body composition estimated using all available CT scan slices.

### Prediction of the stomach and viscera

The results of the current study agree with the findings of Gunderson and Jensen (1987) that at least 10 to 15 scans can predict the volume and subsequently the weight of empty body components with 95% accuracy. The RMSPE for the estimation of stomach volume increased rapidly relative to viscera volume, which is likely because the stomach does not continue throughout the whole body, thus the number of slices involved at equivalent slice intervals is reduced. Additionally, errors of the same magnitude contribute a greater percentage to total volume due to the smaller volume of the stomach relative to the viscera.

The stomach contents in the current study contained a greater proportion of liquid than reports by Bain et al. (2014) and Bond et al. (2017); however, the results were similar to those of De Barbieri et al. (2015). This may have been a result of the differing diets between the studies; although, De Barbieri et al. (2015) and Bond et al. (2017) used similar diets. Lambs in the current study had access to straw prior to CT scanning at the start of the experiment which was not available prior to scans taken at the end of the experiment and yet no differences in proportions of particulate and liquid in the stomach were detected between scanning timepoints. The relationship between the volume and weight of stomach contents estimated by CT scanning is almost identical to that of De Barbieri et al (2015) who reported that postmortem weights of the stomach and contents and those estimated using CT scans were highly correlated (*R*^2^ = 0.92).

### Prediction of empty body composition by treatment

The lower fat tissue mass predicted by the CSIRO model in comparison to the CT scan results for the H2 treatment is possibly explained by the findings of Keogh et al. (2023). Lambs depositing a high proportion of energy as fat have increased efficiency of energy utilization for gain once predicted maintenance energy requirements have been accounted for (Graham, 1980; Keogh et al., 2023). This is due to lower heat production associated with fat deposition compared to lean tissue (Oddy and Sainz, 2002). The efficiency of ME use for protein and fat gain is defined by the slope of the line relating retained energy to ME intake. Values obtained from the literature for the efficiency of protein and fat gain with the least error were respectively 0.4 and 0.7 (Oddy et al., 2024). The efficiency of energy utilization for gain is predicted by the CSIRO model based on the energy density of the diet and doesn’t account for the increasing proportion of fat and increased efficiency of the gain at high levels of feeding and later stages of maturity (Hegarty et al., 1999; Keogh et al., 2023).

The Oddy model similarly predicted lower fat tissue mass than CT fat especially for the H2 treatment. When the Oddy model was evaluated against independent data, similar results were observed for the prediction of fat and it was acknowledged that either heat production was being overestimated or the factor converting fat energy to weight was incorrect (Oddy et al., 2024).

There are potential complications from using CT values to initialize the models and it is not possible to determine whether the model calculations require refinement or the CT scan-derived values for tissue mass are the source of the errors. At the commencement of both feeding periods, there appeared to be carryover effects of the lambs’ prior pasture-based diets despite an 18-d induction period to the pelleted diet in the feedlot pens before the first CT scans. Rumen volume, stomach contents weight, and viscera lean were all large relative to empty body weight particularly at the start of the second feeding period as the lambs were previously on a lower quality pasture supply. Throughout the feeding periods, lambs in all treatments, except H1, either maintained or reduced rumen volume and viscera lean mass, which was as predicted by the Oddy model. The prior pasture-based diet may have resulted in the lambs being leaner for their empty body weight at the start of feeding periods than the lambs from the comparative slaughter studies used to develop the models, which could have affected results. There is a scarcity of data on lambs gaining weight whilst simultaneously decreasing in viscera lean mass; however, CT scanning live lambs may provide an opportunity to collect more of this data and further investigate energy transactions in these circumstances.

It was expected that the lean tissue mass would be overestimated by the CSIRO model particularly for L2 due to the loss in viscera lean observed in this treatment. Rather than predicting viscera lean mass throughout the feeding period as is done by the Oddy model, the CSIRO model-predicts rates of protein and fat gain based on the animal stage of maturity. As there is no input or predicted initial viscera lean mass, the nutritional history of animals is not accounted for. Despite this, predicted lean tissue mass was aligned with CT lean which perhaps highlights potential errors in the designation of lean and fat tissue using the current CT scan analysis methods.

### Potential sources of error by prediction models: SRW

Animal stage of maturity was determined by FFEBW as a proportion of an adjusted SRW in both models. An incorrect SRW could result in prediction errors as a greater SRW increases the proportion of protein and decreases the proportion of fat in the gain at the same FFEBW and *vice versa* when the SRW is reduced (CSIRO, 2007). For the current study, a SRW of 70 kg was used; however, the effects of lower (60 kg) and higher (80 kg) SRW were investigated and these adjustments were unable to compensate for the discrepancies identified using both models.

The under prediction of fat in H2 was marginally improved in both models with a lower SRW as the animal deposited a greater proportion of fat at the same FFEBW. Using a lower SRW the predicted lean tissue mass decreased resulting in inferior predictions of lean tissue mass for all treatments.

The reduction in lean tissue mass with a lower SRW additionally reduced estimated heat production by the Oddy model resulting in increased energy availability for fat deposition. However, the improvement in the prediction of fat tissue mass was minimal, possibly because heat production energy losses due to the initial viscera lean and NVEB mass remain unchanged irrespective of an altered SRW. An increased SRW resulted in lower values for predicted fat tissue mass and increased values for predicted lean tissue mass by both models.

### Potential sources of error by prediction models: viscera lean mass

The Oddy model predicts a target viscera lean mass based on current nutritional information and the initial value is then gradually adjusted throughout the feeding period dependent upon an updated value for target visceral mass. Due to the difficulty in manually editing CT scans to remove intestinal contents, CT viscera lean mass is likely higher than the reality at the commencement of the feeding periods. The initial viscera lean mass will lead to a reduction in estimated fat deposition, as there is less energy available due to increased heat production whilst the lean tissue mass value gradually adjusts to the target viscera mass.

The weight of intestinal contents has shown to be approximately equal to 34% (range; 24 to 38%) of the weight of stomach contents (including reticulo-rumen, abomasum, and omasum) using data from previous studies (De Barbieri et al., 2015; Dougherty et al., 2022). This would result in a reduction in visceral lean mass from the currently estimated values by approximately 1 to 2 kg. However, the weight of intestinal contents appears to differ due to diet type and feeding level. It is also possible that the manual image editing to remove the rumen contents results in the removal of stomach tissue which then contributes to stomach contents weight and somewhat offsets the inability to remove intestinal contents. The weight of stomach tissue is approximately equal to 1.0 to 1.5 kg using data from Dougherty et al. (2022); however, it is unlikely that a large proportion of this tissue is removed in the image editing process.

The current data highlights that observed values at the end of the feeding periods for viscera lean mass are greater than those predicted by the Oddy model with the differences being greater for the high feeding-level treatments. This highlights that to further facilitate the use of CT scans to inform the Oddy model, comparative slaughter studies are required to develop an accurate method for the prediction of stomach-fill and intestinal contents mass which can account for differing diet composition and feeding levels.

### Other potential sources of error by prediction models

The energy density of the diet used in both models was based on the results of the digestibility trial reported by Keogh et al. (2023). The assumed energy density of the pelleted diet used as an input for both models was 0.6 MJ ME/kg DM lower for H2 lambs compared with L2. Increasing the energy density of the diet for H2 lambs improved predictions of fat tissue mass using the CSIRO model; however, predicted lean tissue mass also increased and was greater than CT lean values. In comparison, changes to predicted lean and fat tissue mass were relatively minor when the energy density of the diet was increased using the Oddy model. This indicates that the increase in available energy for tissue deposition by increasing ME intake was greater for the CSIRO model than the Oddy model. This suggests that when using the Oddy model, less fat is being deposited because the heat production being generated from viscera lean mass is greater than what actually occurs, possibly due to the input CT scan values used to initialize the model. Irrespective, increasing the energy density of the diet demonstrated that both models are susceptible to errors if the input energy density of the diet or level of feeding is inaccurate.

Lambs were fed at a restricted feeding level in pens of 3. This likely resulted in some lambs within pens consuming quantities different to the average allocated per lamb. Lambs within pens consuming different quantities of feed will also have contrasting viscera lean tissue mass which would have altered the energy availability for tissue gain and the resultant composition of gain. Whilst much of the between-animal variation within pens would be offset as the analysis of prediction errors was conducted on a per-pen basis, it would have been beneficial to analyze tissue gain on individuals with known individual animal intake.

Additionally, it is possible that there is some error in the assignment of different pixels and pixel densities to lean and fat tissue and gut fill components which could result in the overestimation of one tissue mass and a corresponding underestimation of the alternate tissue (Alston et al., 2004). Cumulative errors would then occur as estimates of some masses are reliant on others (i.e., NVEB tissue mass is reliant on calculations of visceral tissue mass and the calculated FFEBW, which is calculated using an estimated stomach content mass).

### Implications and Conclusion

The use of CT scanning of individual animals provides an opportunity to analyze body compositional changes at multiple timepoints which will facilitate the development of more precise ruminant growth models. The *Cavalieri method* and representative scan sites both show potential to accurately assess body composition; however, the *Cavalieri method* proved advantageous for the analysis of composition of the viscera due to the ability to estimate both stomach and viscera volumes. Estimated empty body tissue weights using CT scans correspond well with predicted tissue mass using 2 ruminant growth models; however, it is likely that the inability to manually dissect all intestinal contents using CT scans results in an overestimation of viscera lean tissue mass. Further verification is needed by combining CT scanning and comparative slaughter, especially with respect to the weight of viscera tissues and stomach and intestinal contents.

Predicted lean tissue mass using both models was closely aligned with lean tissue mass estimated using CT scans irrespective of feeding level and stage of maturity. This highlights both the utility of the predictive models and the value of CT scanning to estimate body composition. Further investigation of energy transactions in growing lambs fed diverse and changing diet types is necessary to better predict empty body composition and further develop predictive growth models. The model described by Oddy which can better account for the effects of diet type, feeding level, stage of maturity, and genotype on empty body composition shows promise but requires more work.

CT scanning should be incorporated into live animal feeding experiments designed to investigate body composition on energy balance where possible as it provides greater precision than measuring LW alone. Both models predicted lower fat tissue mass than the CT values, particularly in period 2, indicating that either the distribution of tissues to empty body components using CT scans requires further evaluation, particularly in the viscera, or that the efficiency of energy utilization by different tissues within the models needs further refinement. CT scans enable the simplified measurement of body composition in lambs; however, the comparison with the Oddy model highlighted the need to evaluate the accuracy of the collected data by comparative slaughter.

## Abbreviations

CSU: Charles Sturt University
CT: computerized tomography
DM: dry matter
FFEBW: fleece-free empty body weight
LW: liveweight
ME: metabolizable energy
MSPE: mean square prediction error
NVEB: fat-free non-viscera empty body
PDV: portal drained viscera
RMSPE: root mean square prediction error
SRW: standard reference weight
